# Supplementary material for: Prognostic Relevance of RIP140 and ERβ Expression in Unifocal Versus Multifocal Breast Cancers: A Preliminary Report
Source: Int J Mol Sci. 2019 Jan 18;20(2):418. doi: 10.3390/ijms20020418 (PMC6359229; doi:10.3390/ijms20020418)
Supplement: Supplementary file 1 [file ijms-20-00418-s001.pdf]

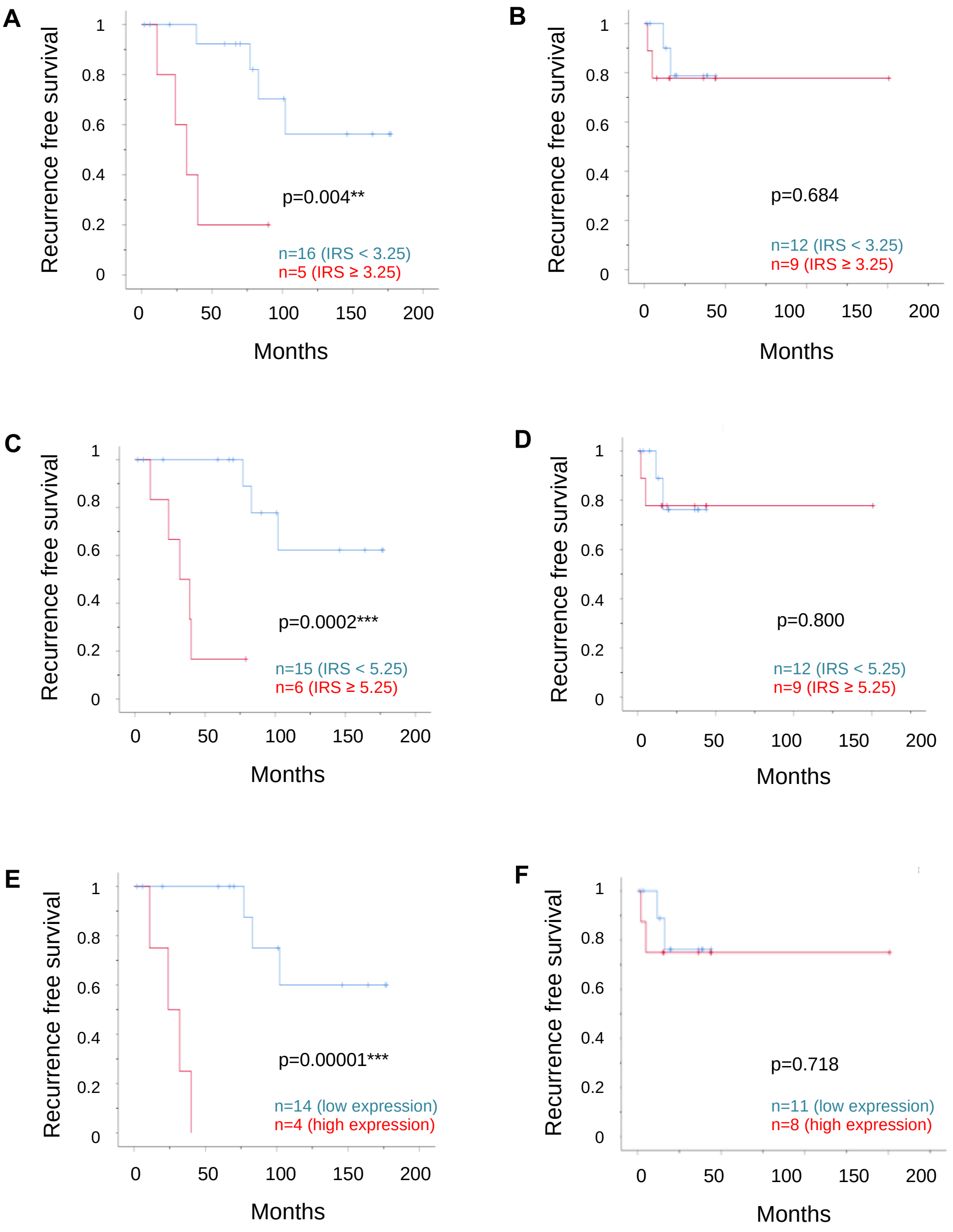

Figure S1. Recurrence free survival (RFS) according to the expression of RIP140 and ERβ, alone or combined. Kaplan–Meier analysis of the correlation between low vs. high expression of two markers with RFS in the matched pairs (unifocal for **A**, **C**, **E** and multifocal for **B**, **D**, **F**). The analysis was performed with RIP140 (**A**,**B**) or ERβ (**C**,**D**) expression, or with RIP140 expression combined with ERβ expression (**E**,**F**). Correlations are statistically significant for $p < 0.01$ (**) and for $p < 0.001$ (***).
